# Regional variability in the associations between social and health-related risk factors and memory across Europe

**DOI:** 10.1038/s41598-026-56180-7

**Published:** 2026-06-06

**Authors:** Huixia Savannah Wang, Anna Rieckmann, Maria Josefsson

**Affiliations:** 1 Department of Statistics, Umeå School of Business, Economics and Statistics, Umeå University, Umeå, Sweden; 2 Institute of Psychology, Universität der Bundeswehr München, München, Germany; 3 Department of Diagnostics and Intervention, Umeå University, Umeå, Sweden

**Keywords:** Memory, SHARE, Bayesian additive regression trees, Multilevel modeling, Diseases, Health care, Medical research, Risk factors

## Abstract

Interventions targeting social and health-related risk factors are thought to reduce the risk of cognitive decline and dementia in older age. Despite well-known social, economic, and cultural differences across European countries, little is known about how these factors influence associations with memory function in different geographical contexts. This study examined the relationship between five social and health-related risk factors, namely living alone, physical inactivity, obesity, depression, and cardiometabolic and cardiovascular conditions, and memory function across Europe. Data came from the Survey of Health, Ageing, and Retirement in Europe (SHARE), a cross-national study of older adults. The sample included cross-sectional data for 102,851 adults aged 50–102 years from 20 European countries, grouped into four regions: Northern, Western, Eastern, and Southern Europe. Memory function was assessed using a sum score of immediate and delayed recall tests. A flexible Bayesian machine learning approach for multilevel data was applied to assess heterogeneity of associations in the total sample and in analyses stratified by education and age. All five social and health-related risk factors were negatively associated with memory overall, but the strength and, for some factors, the direction of these associations varied across regions. In particular, the associations for living alone, obesity, and physical inactivity differed between Eastern and Southern Europe compared with Northern and Western Europe. These findings highlight substantial geographical heterogeneity in the associations between social and health-related risk factors and memory, which should be considered when designing and implementing public health interventions.

## Introduction

Europe is faced with the challenge of a rapidly aging population, a demographic shift that is anticipated to almost double the number of people living with dementia by the year 2050^1^. It has been estimated that as much as 40% of Alzheimer’s and related dementia cases may be influenced by modifiable factors^2^, and that a favorable social and health-related risk-factor profiles might even counteract a genetic risk for Alzheimer’s disease^3–5^. This presents a promising opportunity for interventions targeting social and health-related risk factors to mitigate the risk of cognitive decline and dementia in older age. Nevertheless, given the social, economic, and cultural differences across European countries, it is plausible that such interventions may need to be tailored accordingly to effectively target cognitive health disparities.

Significant disparities in cognitive performance and rates of decline are evident among older adults across various European regions. Specifically, southern and eastern European countries exhibit higher prevalence of cognitive impairment compared to their western and northern counterparts^6^. Longitudinal studies have moreover shown that the northern countries initially displayed the highest cognitive performance but experienced greater decline over time, while Mediterranean countries showed the reverse pattern, and western and central/eastern countries demonstrated intermediate performances^7–9^.

Current evidence indicates that cognitive performance in older adults is influenced by several modifiable social and health-related risk factors, spanning from behavioral and social conditions to environmental influences^10–13^. Studies comparing risk factor associations across multiple countries have revealed significant differences, including between South Korea and the United States (U.S.)^14^, South Korea and Japan^15^, and among North American^16^ and Latin American countries^17^. Sociodemographic factors such as age, gender, and education were linked to cognition in most regions, whereas sociobehavioral factors, including obesity, social support, hospitalization^15^, depression, high blood pressure, and diabetes^14^, were associated with cognitive impairment in Japan and the U.S., but not in South Korea. The proportion of dementia cases attributable to 12 modifiable social and health-related risk factors also varied significantly across Latin America^17^. Despite geographical distance, Canada’s risk factor profile closely resembled that of Australia and Denmark^16^. These findings underscore substantial cross-national differences in how social and health-related risk factors influence cognitive performance. However, few studies have examined these associations in European populations.

Although the reasons for the observed heterogeneity in these associations are not yet fully understood, the findings highlight important regional differences in which social and health-related risk factors may be most urgent to address. Therefore, this study aims to investigate the association between five social and health-related risk factors and memory function across European populations.

To address this research question, we employ a statistical model for multilevel data that integrates Bayesian machine learning^18^. While multilevel linear regression is a powerful and widely used statistical technique for modeling cross-national data^7,9,14^, it may be less suitable in settings with non-linear relationships and complex interactions. Such challenges are well recognized in aging research^19–21^. The model can be conceptualized as a flexible approach that allows complex relationships to be modeled while still providing interpretable estimates.

## Methods

### Ethics approval

All methods were carried out in accordance with relevant guidelines and regulations. The use of data from the Survey of Health, Ageing and Retirement in Europe (SHARE) complies with the Survey of Health, Ageing and Retirement in Europe – European Research Infrastructure Consortium (SHARE-ERIC) Conditions of Use and adheres to the principles outlined in the Declaration of Helsinki. All participants provided written informed consent, and their data were pseudonymized. Participants were informed about data storage, usage, and their right to withdraw consent. The current study received approval from the Swedish Ethical Review Authority (Dnr: 2022-06094-01).

### Study design and research participants

Data were drawn from SHARE, a cross-national study of older adults in European countries and Israel^22^. For this study, we considered data from the respondents’ baseline assessment. The study includes respondents aged 50 years or older without self-reported dementia. A total of 114,230 respondents were selected from SHARE waves 1–2 and 4–6. Of these, n = 8,303 participants were excluded due to missing values for any of the study variables (see below). In addition, to ensure comparability within a European context, Israel was excluded from further analyses (n = 3,076).

The final dataset includes cross-sectional information (data collected at the enrollment wave) on 102,851 European adults aged 50 to 102 years from 20 countries. Countries were grouped into four regions according to the United Nations Statistics Division classification^23^: Northern Europe (Sweden, Denmark, Estonia, Ireland), Western Europe (Austria, Germany, Netherlands, Switzerland, Belgium, Luxembourg, France), Southern Europe (Croatia, Spain, Portugal, Slovenia, Italy, Greece), and Eastern Europe (Czech Republic, Poland, Hungary).

### Study variables

*Outcome* Memory function was assessed using a composite score based on immediate and delayed word recall tests administered as part of the SHARE survey. Respondents were asked to recall a list of words immediately after presentation and again after a delay, capturing key aspects of episodic memory. The assessments were conducted during standardized computer-assisted personal interviews (CAPI). The total memory score ranges from 0 to 20, with higher scores indicating better memory performance.

*Primary exposures* We examined five primary exposures reflecting social and health-related risk factors and cardiovascular morbidity: obesity, physical inactivity, living alone, depression, and a composite indicator of cardiometabolic and cardiovascular conditions (CMC). All variables were treated as binary indicators.

Obesity was defined as a body mass index (BMI) greater than 30, calculated from self-reported height and weight. Physical inactivity was defined based on self-reported frequency of engaging in vigorous physical activities (e.g., sports, heavy housework, or labor-intensive work), with individuals reporting such activities less than once a week classified as physically inactive. Living alone was defined based on household size, with single-person households classified as living alone. Depression was assessed using the EURO-D scale, with scores greater than 3 (range 0–12) indicating clinically relevant depressive symptoms.

The CMC indicator was constructed based on self-reported diagnoses of hypertension, diabetes, high cholesterol, heart disease, and stroke. Hypertension, diabetes, and high cholesterol represent cardiometabolic risk factors, while heart disease and stroke reflect established cardiovascular conditions. A binary indicator was defined taking the value 1 if an individual reported at least one of these conditions, and 0 otherwise.

*Covariates* Models were adjusted for sex, age, education, activities of daily living (ADL), and enrollment test wave. These covariates were selected based on prior literature and their potential role as confounders of the association between social and health-related risk factors and memory function.

Education was assessed using the International Standard Classification of Education (ISCED-97), ranging from 0 (none/early childhood education) to 6 (doctoral or equivalent level). Education was categorized into three groups: low (ISCED 0–2), medium (ISCED 3–4), and high (ISCED 5–6). Individuals with unknown or ongoing education were classified as “other”.

The ADL index reflects the number of limitations in activities of daily living^24^, including dressing, walking, grooming, eating, transferring, and toileting, and ranges from 0 to 6. The ADL index was included as a continuous variable.

Details of variable measurement, coding, analytic role, and rationale are provided in Supplementary Table S1.

### Statistical analysis

This study investigates associations between social and health-related risk factors and memory function across 20 European countries and four regions. Given the multilevel structure of the data, where individuals within countries may share common characteristics, and the potential for non-linear associations, we used a Bayesian machine learning approach, General Bayesian Additive Regression Trees (GBART)^18^.

Compared to conventional linear mixed models (LMMs), which require explicit specification of model structure and interactions, GBART provides a more flexible framework for modeling associations. In settings where relationships may be non-linear and involve complex interactions, pre-specifying such structures can lead to model misspecification. In brief, GBART learns patterns directly from the data rather than relying on a pre-specified functional form, allowing non-linear relationships and interactions between variables to be captured without strong parametric assumptions.

To account for between-country heterogeneity, we allowed the association between each social and health-related risk factor and memory to vary across countries by including exposure–country interactions, yielding country-specific estimates. In this way, the effect of each risk factor is allowed to differ between countries rather than assuming a common effect across all populations. A detailed description of the model specification is provided in Supplementary Information Appendix 1.

To maintain interpretability and avoid overly complex model specifications, we estimated separate models for each risk factor. In each model, the focal risk factor was treated as the exposure of interest, while the remaining risk factors and relevant covariates (age, sex, education, activities of daily living (ADLs), and enrollment wave) were included as adjustment variables to account for potential confounding. This approach allows us to isolate the effect of each risk factor on memory while controlling for other exposures.

In addition to estimating country-specific effects, we also examined (a) the overall association across all countries and (b) associations within broader European regions. These were obtained using an inverse-variance approach, commonly applied in meta-analysis, to derive pooled estimates across countries (see Supplementary Information Appendix 2).

We further explored potential variation in associations by age and education through stratified analyses. Age stratification (< 65 vs $\ge$ 65 years) was chosen based on prior literature in cognitive aging, where episodic memory performance is often relatively stable until approximately 60–65 years of age, followed by a more pronounced decline^25^. Participants were therefore divided into two age groups: younger (< 65 years) and older ($\ge$ 65 years). In addition, the sample was categorized into two education groups (low vs. medium/high education). Individuals with unknown or ongoing education status were excluded from these analyses (approximately 1% of the sample).

All analyses were conducted using the *SoftBart* package in R (version 4.1.1) at the high-performance computing infrastructure NAISS-SENS^26^. Default model settings were used unless otherwise specified (see Supplementary Information Appendix 1). We obtained 6,000 posterior samples for inference after discarding an initial burn-in period. Convergence was assessed using trace plots.

Associations between social and health-related risk factors and memory were summarized using 95% credible intervals (CI). Associations were considered statistically meaningful if the corresponding credible interval did not include zero.

To describe sample characteristics and regional differences, analysis of variance (ANOVA) was used for continuous variables (memory and age), and Pearson’s chi-squared test was used for categorical variables.

## Results

**Table 1:** Baseline characteristics of the SHARE data across regions.

	East	North	South	West	*p* Value
	(n=13 103)	(n=19 059)	(n=29 200)	(n=41 489)	
Memory	9 ± 3	9 ± 4	8 ± 4	9 ± 4	$<.001$
(min, max)	(0, 20)	(0, 20)	(0, 20)	(0, 20)	
Background characteristics					
Age	64 ± 9	64 ± 10	64 ± 10	63 ± 10	$<.001$
(min, max)	(50, 100)	(50, 101)	(50, 102)	(50, 100)	
Male	5 733 (44%)	8 579 (45%)	13 566 (46%)	19 229 (46%)	$<.001$
ADLs > 0	1 513 (12%)	1 892 (10%)	2 559 (9%)	3 807 (9%)	$<.001$
Education level					
high	1 612 (12%)	5 591 (29%)	3 727 (13%)	10 135 (24%)	$<.001$
medium	6 167 (47%)	7 502 (39%)	7 626 (26%)	16 676 (40%)	$<.001$
low	5 259 (40%)	5 887 (31%)	17 729 (61%)	14 405 (35%)	$<.001$
others	65 (0%)	79 (0%)	118 (0%)	273 (1%)	$<.001$
Social and Health-related Risk Factors					
CMC	8 071 (62%)	9 996 (52%)	16 197 (55%)	21 216 (51%)	$<.001$
Hypertension	6172 (47%)	7131 (37%)	10924 (37%)	13284 (32%)	$<.001$
Diabetes	1975 (15%)	1729 (9%)	3545 (12%)	3979 (10%)	$<.001$
Cholesterol	2725 (21%)	3649 (19%)	6940 (24%)	9252 (22%)	$<.001$
Heart disease	2209 (17%)	2809 (15%)	3184 (11%)	4595 (11%)	$<.001$
Stroke	646 (5%)	964 (5%)	823 (3%)	1433 (3%)	$<.001$
Living alone	2 514 (19%)	3 996 (21%)	4 306 (15%)	8 698 (21%)	$<.001$
Physical inactivity	6 193 (47%)	6 657 (35%)	12 697 (43%)	16 252 (39%)	$<.001$
Depression	4 105 (31%)	4 787 (25%)	8 498 (29%)	10 117 (24%)	$<.001$
Obesity, BMI > 30	3 626 (28%)	3 848 (20%)	5 973 (20%)	7 545 (18%)	$<.001$

Continuous variables are presented as mean ± SD and dichotomous variables as n ($\%$); SD is standard deviation; ADLs denotes difficulties with daily living activities; CMC denotes the cardiometabolic and cardiovascular risk indicator, showing the presence of one or more of either hypertension, diabetes, cholesterol, heart disease or stroke

The study sample included 102,851 participants, with a mean age of 64 years (SD = 10), of whom 46% were male. Among them, 9,771 individuals (10%) reported difficulties with activities of daily living (ADLs). A substantial proportion of the sample had a low level of education (42%), while 37% had a medium level and 20% had a high level; 1% were classified as “other”.

Regarding social and health-related risk factors, 55,480 respondents (54%) reported at least one cardiometabolic and cardiovascular condition (CMC), including hypertension, diabetes, high cholesterol, heart disease, or stroke. Additionally, 19,514 individuals (19%) reported living alone, 41,799 (41%) were physically inactive, 27,507 (27%) had depressive symptoms (EURO-D $\ge$ 3), and 20,992 (20%) were classified as obese (BMI > 30).

Baseline characteristics across regions are presented in Table 1. Southern and eastern regions exhibited lower education levels compared to northern and western regions. The eastern region showed the highest proportions of respondents with CMC, physical inactivity, depression, and obesity, whereas northern and western regions generally had lower prevalence of these risk factors. The southern region had the lowest proportion of individuals living alone and also the lowest mean memory score, while the remaining regions showed similar levels of memory performance.

**Fig. 1:**
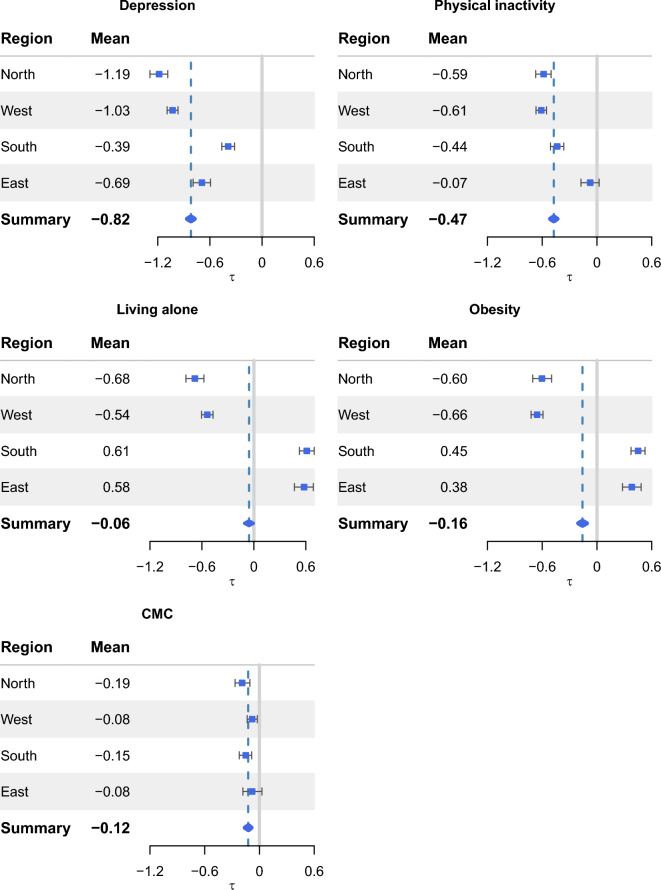
Estimated the associations and the corresponding credible intervals (CIs) for the five social and health-related risk factors, across regions and overall pooled associations (marked as *summary*). The solid vertical grey lines denote zero (i.e., no association), and the blue vertical lines denote the pooled overall associations.

Figure 1 and Supplementary Table S2 presented the pooled associations, overall and across regions, illustrating posterior means and credible intervals (CIs) of the social and health-related risk-factor associations. The estimated differences reflected absolute changes in the number of correctly recalled words on the 0–20 memory scale.

In the overall sample, individuals exposed to any of the five social and health-related risk factors exhibited lower memory scores compared to those not exposed, after adjusting for age, sex, education level, activities of daily living (ADL), enrollment test wave, and the other risk factors. Depression and physical inactivity demonstrated the most pronounced associations. For example, depression was associated with a difference of −0.82 points in memory score (95% CI −0.86 to −0.77), and physical inactivity with a difference of −0.47 points (95% CI −0.51 to −0.43). The remaining risk factors (living alone, obesity, and CMC) were also associated with lower memory scores, although with smaller effect sizes.

**Fig. 2:**
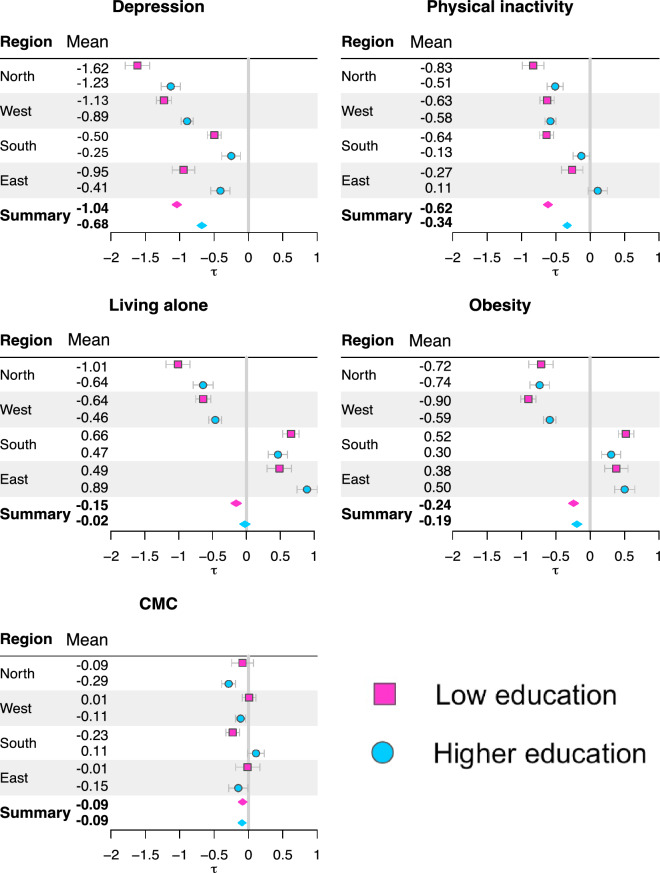
Education-stratified analyses. Estimated associations and the corresponding credible intervals (CIs) for the five social and health-related risk factors, across regions and overall pooled associations (marked as *summary*). The solid vertical grey lines denote zero (i.e., no association), and the blue vertical lines denote the pooled overall associations.

Education-stratified analyses (Fig. 2 and Supplementary Table S3) showed that individuals with lower education experienced more pronounced negative associations compared to those with higher education, except for CMC, where associations were small and similar across groups.

**Fig. 3:**
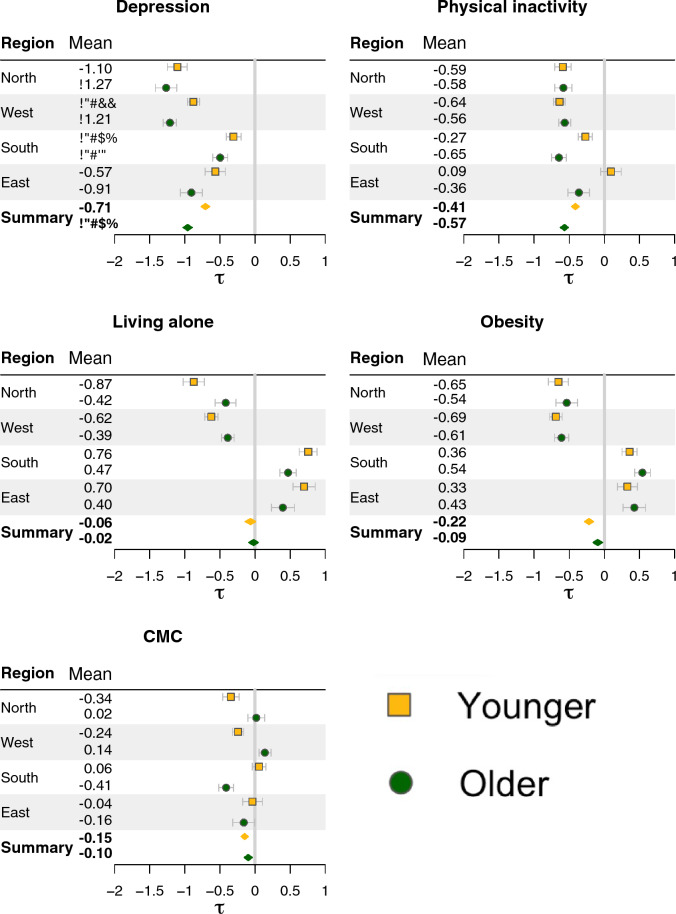
Age-stratified analyses. Estimated associations and the corresponding credible intervals (CIs) for the five social and health-related risk factors, across regions and overall pooled associations (marked as *summary*). The solid vertical grey lines denote zero (i.e., no association), and the blue vertical lines denote the pooled overall associations.

Age-stratified analyses (Fig. 3 and Supplementary Table S4) showed that depression and physical inactivity were more strongly associated with memory among older adults ($\ge$ 65 years) compared to younger individuals (< 65 years). In contrast, associations for CMC and obesity were less pronounced in older adults. Living alone showed small, non-significant associations in both age groups.

Regional differences were observed (Fig. 1). Northern and western regions showed stronger associations between depression and memory compared to southern and eastern regions. Physical inactivity showed a weaker and non-significant association in the eastern region compared to other regions. Living alone and obesity were positively associated with memory in southern and eastern regions but negatively associated in northern and western regions. Only minor regional differences were observed for CMC.

In Fig. S1 shows country-level results. Countries within regions were largely clustered for depression, physical inactivity, obesity, and living alone, whereas more heterogeneous patterns were observed for CMC.

In education-stratified analyses, all regions showed stronger negative associations of depression among individuals with low education. Physical inactivity showed stronger associations among low-educated individuals in most regions, although non-significant in highly educated individuals in the eastern region. No clear differences were observed in the western region.

For CMC, associations were generally small. Among individuals with low education, associations were only significant in the southern region. Among highly educated individuals, associations were non-significant and in some cases positive in the southern region.

Age-stratified analyses revealed additional regional differences. In northern and western regions, living alone showed stronger negative associations among younger individuals compared to older, whereas the opposite pattern was observed in southern and eastern regions. Physical inactivity showed stronger negative associations among older individuals in southern and eastern regions, while associations were similar across age groups in northern and western regions. For CMC, associations were negative among younger individuals and non-significant or positive among older individuals in northern and western regions, whereas the opposite pattern was observed in southern and eastern regions.

## Discussion

The present study aimed to improve understanding of the association between social and health-related risk factors and memory functioning in a European setting. By incorporating a flexible machine learning technique for multilevel data^18,27^, we explored variation in these associations across diverse geographical contexts. Although the overall analyses showed negative associations between all examined social and health-related risk factors and memory, substantial regional variation was observed in the magnitude of these associations and, for some factors, even in their direction. These findings support the presence of heterogeneity in risk-factor associations across Europe and should be taken into account when designing and implementing public health interventions.

Previous research has shown that depression is associated with deficits in episodic memory and broader cognitive functioning^28,29^. Our findings replicate and extend these results by demonstrating a strong association between depression and poorer memory performance across all European regions. The stronger association of depression with memory among older individuals and those with lower education levels may be explained by age-related neurological and cognitive vulnerabilities, such as increased hippocampal atrophy, a cumulative burden of depression in older age, and reduced cognitive reserve^30,31^. Additionally, memory deficits in depression may be mistaken for early signs of dementia^32,33^, which could contribute to the stronger associations observed among older participants.

We found a weak overall association between living alone and poorer memory performance. Similar findings have been reported in some previous studies^34–36^, but not all^37,38^. The association between living arrangements and memory function varied substantially between Northern/Western and Eastern/Southern European regions. While living alone was negatively associated with memory in Northern and Western Europe, it showed a positive association in Eastern and Southern Europe. This regional discrepancy may reflect differences in cultural norms, healthcare access, financial support systems, and social structures^39–41^. In Northern and Western Europe, extensive home-care services and financial independence may allow older adults to live alone even in the context of declining health. However, living alone is also strongly linked to social isolation, a known risk factor for mental health problems, cardiovascular disease, and mortality^42–44^, which may help explain the negative association observed in these regions. In contrast, in Eastern and Southern Europe, multigenerational households are more common and family members often provide care for older relatives. Living alone in these settings may therefore reflect a particularly healthy and independent subgroup rather than a risk for cognitive decline. This suggests that the association between living arrangements and cognitive function is context-dependent and shaped by broader social and policy environments.

Our findings also revealed regional discrepancies in the association between obesity and memory function. Prior research suggests that the association between obesity and socioeconomic status (SES) differs by country^45–48^. In Northern and Western Europe, obesity is often associated with lower SES, which in turn is linked to poorer diet quality, metabolic conditions, and reduced health awareness, all of which may contribute to its negative association with memory function. In contrast, in Eastern and Southern Europe, obesity may be more common among individuals with higher SES, who generally have better access to healthcare, nutrition, and cognitive stimulation, potentially explaining the observed positive associations. In Southern Europe, adherence to a traditional Mediterranean diet, even among individuals with obesity, may also mitigate some adverse cognitive effects through its favorable fat composition and anti-inflammatory properties^49^. The pattern observed in Eastern and Southern Europe also aligns with the “obesity paradox,” whereby excess weight, particularly in older populations, has sometimes been linked to protective effects for mental and physical health^50,51^, while weight loss is associated with illness and cognitive impairment^52^. These findings highlight the importance of considering socioeconomic, health, and dietary factors when examining the relationship between obesity and cognitive function across different contexts, particularly in settings undergoing rapid economic and epidemiological transitions^53^.

Previous research has shown that physical inactivity is associated with poorer cognitive performance and an increased risk of memory decline^54,55^. Our findings are consistent with this, showing a strong association between physical inactivity and poorer memory performance, particularly among older adults and individuals with lower education levels. Regional differences were observed, with a weaker association in Southern and Eastern regions among younger individuals and those with higher education levels, mirroring trends observed for obesity. Although the precise mechanisms remain unclear, physical inactivity and memory deficits in older adults may be linked through shared pathways, such as cardiovascular dysfunction or systemic inflammation.

Previous cross-sectional and longitudinal studies have shown that cardiometabolic and cardiovascular conditions are associated with poorer cognitive performance and memory decline^56–58^. Consistent with this, we observed a moderate overall association between CMC and memory function. However, substantial regional heterogeneity was observed, particularly in the stratified analyses. In Northern and Western Europe, the negative association between CMC and memory function was more pronounced among younger individuals with higher education levels, a pattern also reported in epidemiological studies^21,59^. Rather than reflecting a particular vulnerability of this group, this may indicate the influence of well-developed welfare and healthcare systems in these regions^60^, which prioritize preventive cardiovascular care from middle age onward. In contrast, in Southern and Eastern Europe, cardiovascular conditions may remain undetected until later life, where a greater emphasis is placed on acute treatment^61^. This may lead to a greater cumulative burden of cardiovascular morbidity that manifests more strongly in older age. Consistent with this interpretation, we observed a reversed age pattern in these regions, with stronger associations between CMC and memory among older individuals. Taken together, these findings highlight the interplay between cardiovascular health, healthcare systems, and cognitive function, and underscore the importance of population-level preventive strategies across the lifespan.

There are limitations to consider. First, the cross-sectional nature of this study provides only a snapshot of the relationship between the five social and health-related risk factors and memory function, rather than a dynamic view. This design limits the ability to establish causality. Therefore, longitudinal studies are necessary to confirm these findings and track changes over time.

Furthermore, despite adjustments for several covariates using a flexible modeling technique, residual confounding from unmeasured variables, such as genetic predispositions, unrecognized comorbidities, or socioeconomic factors, may remain. In addition, the binary CMC indicator captures only the presence of any cardiometabolic or cardiovascular condition and does not reflect cumulative burden or dose–response effects. As a result, individuals with one condition are treated the same as those with multiple conditions. Future work could consider more detailed representations, such as summed indices or continuous measures.

The questionnaire relies heavily on self-reported data, which are known to be prone to recall bias and social desirability bias, potentially affecting data accuracy. There are also likely differences among countries in the detection and diagnosis of cardiometabolic and cardiovascular conditions. As such, these conditions may be more frequently undetected, rather than truly absent, in some countries, particularly among younger individuals. Consequently, the negative long-term effects might be underestimated, and efforts should focus on identifying these high-risk groups.

Finally, participation in the survey may have been influenced by selection bias, with healthier or more cognitively intact individuals more likely to respond, potentially skewing the results.

In conclusion, the present study revealed negative associations between five social and health-related risk factors and memory performance. However, the magnitude of these associations, and for some factors even their direction, varied across European regions. Clear differences were observed between Eastern/Southern and Northern/Western Europe, which may reflect broader social, cultural, economic, and nutritional differences. These findings support the notion of heterogeneity in risk-factor associations across Europe and should be taken into account when planning and implementing public health interventions.

## Supplementary Information

Supplementary Information.

## Data Availability

The data that support the findings of this study are available from the Survey of Health, Ageing and Retirement in Europe (SHARE, www.share-project.org), but restrictions apply to the availability of these data, which were used under license for the current study and are therefore not publicly available. Data are however available from the authors upon reasonable request and with permission of SHARE-ERIC.
