# Supplementary material for: Regional variability in the associations between social and health-related risk factors and memory across Europe
Source: Sci Rep. 2026 Jun 6;16:17531. doi: 10.1038/s41598-026-56180-7 (PMC13242514; doi:10.1038/s41598-026-56180-7)
Supplement: Supplementary file 1 — Supplementary Information. [file 41598_2026_56180_MOESM1_ESM.pdf]

Supplement Information for Regional Variability in the Associations Between Social and Health-related Risk Factors and Memory Across Europe

Huixia Savannah Wang, MSc[1], Anna Rieckmann, PhD[2,3] and Maria Josefsson, PhD[1]

[1]Department of Statistics, Umeå School of Business, Economics and Statistics, Umeå University, Umeå, Sweden.
[2]Institute of Psychology, Universität der Bundeswehr München, München, Germany.
[3]Department of Diagnostics and Intervention, Umeå University, Umeå, Sweden.

Corresponding author(s): Huixia Savannah Wang (huixia.wang@umu.se)

Appendix 1

Details of the Bayesian Semiparametric Multilevel Model

This study aims to investigate associations between five lifestyle risk factors and memory function across 20 European countries and four regions. Given the multilevel nature of the data - where individuals within countries share common characteristics and are therefore correlated - and the potentially complex associations between our predictors and the outcome, we employ a novel Bayesian machine learning method known as the General Bayesian Additive Regression Tree (GBART) model[1]. Specifically, the GBART model, similarly as to the linear multilevel model[2], incorporates one or more grouping variables, such as countries, as random effects. However, rather than incorporating the predictors as fixed effects through a linear model specification, GBART models the association between the predictors and the outcome using Bayesian machine learning (i.e., Bayesian Additive Regression Trees[3]), which are less prone to biased estimates due to model misspecification. In particular, we consider the following model

$$y_{ik} = r(x_{ik}) + \beta_k z_{ik} + \epsilon_{ik} \quad (1)$$

where y_{ik} is the memory outcome for individual $i = 1,..., n_k$ in country $k = 1,...,K$. The covariate vector x_{ik} contains relevant predictors, and the function $r(x_{ik})$ is a (unknown) function describing relationships in predictors with our outcome, which is modeled using *soft* Bayesian additive regression trees[4]. The second term on the right-hand-side in (1) includes the random effects which are modeled as a linear extension. Here, the variable z_{ik} indicates whether an individual was exposed to a specific lifestyle risk factor ($z_{ik} = 0.5$) or not ($z_{ik} = -0.5$) , for all other participants $z_{ik} = 0$. To improve numerical stability in the computations and interpretability of the coefficients we use the -0.5/0.5 coding instead of the more common 0/1 coding. As such, the random effects parameters, β_k, represents the difference in memory between the exposed and unexposed groups in country k. Hence, we allow the risk factor effects on memory to vary between countries. In addtion, the error term $\epsilon_{ik} \sim N(0,\sigma^2)$. This model is implemented the *gsoftbart_regression* function in the *Softbart* package for R[5]. Note that we fit separate models for each lifestyle risk factor, and that the other risk factors are included as covariates in x_{ik} along with the other predictors (i.e. sex, age, education, ADLs and the respondent's enrollment test wave).

Appendix 2

Estimation of the pooled overall effects

In addition to estimating country-specific memory effects, β_k, we are also interested in: i) the overall effect across all countries and ii) pooled effects for different European regions. These effects are computed by using the inverse variance approach commonly used in meta-analyses[6]. In particular, the pooled effect is calculated as a weighted average of the country-specific estimates, $\hat{\beta}_k$, as follows

$$\sum_{k=1}^{K} \hat{\beta}_k \frac{w_k}{\sum_{k=1}^{K} w_k}$$

The inverse variance approach assigns weights, w_1, ..., w_K, to each country based on the inverse of its variance, giving more weight to results from countries with higher precision (i.e., lower variance) and vice versa. Here, the method incorporate two variance components in the weights; a within-country variance component, Var($\hat{\beta}_k$), and a between-country variance component, τ^2. That is,

$$w_k = \frac{1}{\mathrm{Var}(\hat{\beta}_k) + \tau^2}$$

The estimated country-specific effect $\hat{\beta}_k$ is obtained by averaging over the $m = 1, ..., M$ posterior samples. Similarly, the sampling standard deviation, $\sqrt{Var(\hat{\beta}_k^{(m)})}$, is obtained by computing the standard deviation of the posterior samples $\beta_k^{(m)}$ for all m. The between-country variance, $\hat{\tau}$, is obtained by instead computing the standard deviation of country-specific effects $\hat{\beta}_k$ for all k.

Appendix 3

Supplementary Figure S1 Estimated associations of five lifestyle risk factors across the European countries.The solid grey line denotes the zero (i.e. no association), and solid blue line is the pooled association

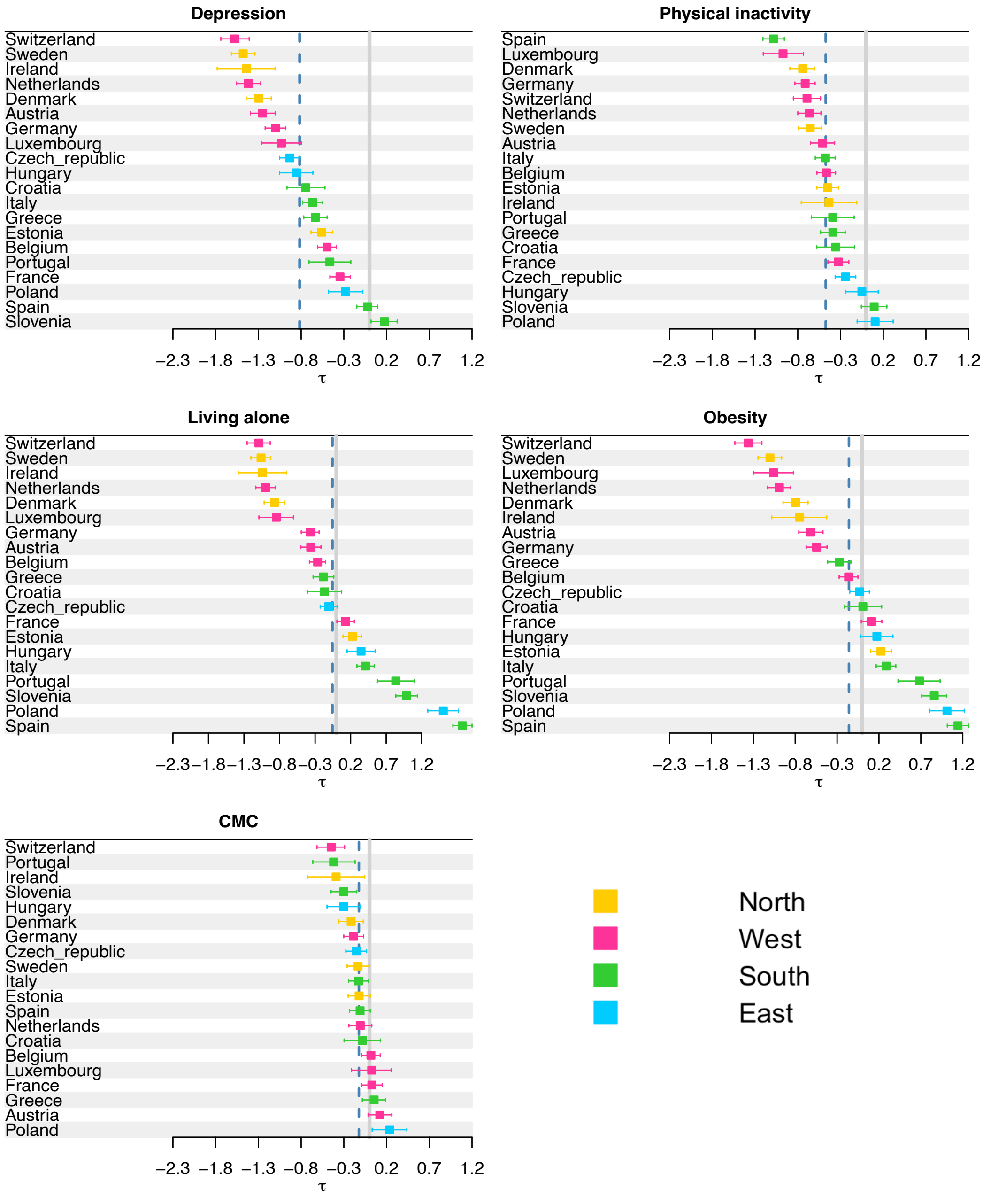

The solid vertical grey lines denotes zero (i.e., no effect); the blue vertical lines the average of pooled overall effects.

Appendix 4

Supplementary Table S1. Measurement, coding, and analytic role of study variables

Variable	Measurement	Coding	Role	Short rationale
Memory function	Assessed during standardized computer-assisted personal interviews (CAPI) in SHARE using immediate and delayed word recall tests.	Composite episodic memory score ranging from 0 to 20, obtained by summing immediate and delayed recall scores; higher values indicate better memory performance.	Outcome	Captures overall episodic memory performance, the main cognitive domain of interest.
Obesity	Derived from self-reported height and weight collected in SHARE.	1 = BMI > 30; 0 = BMI ≤ 30.	Primary exposure	Included as a social and health-related risk factor potentially associated with memory function.
Physical inactivity	Based on self-reported frequency of engaging in vigorous physical activity (e.g. sports, heavy housework, labor-intensive work).	1 = vigorous physical activity less than once a week; 0 = once a week or more.	Primary exposure	Included because low physical activity is a modifiable risk factor linked to cognitive aging.
Living alone	Derived from reported household size in SHARE.	1 = single-person household; 0 = household size > 1.	Primary exposure	Included as a social risk factor that may reflect reduced social support or social engagement.
Depression	Assessed using the EURO-D scale in SHARE.	1 = EURO-D score > 3; 0 = EURO-D score ≤ 3. EURO-D ranges from 0 to 12.	Primary exposure	Included because depressive symptoms are associated with poorer cognitive functioning and may confound or contribute to memory differences.
Cardiometabolic and cardiovascular conditions (CMC)	Constructed from self-reported doctor diagnoses of hypertension, diabetes, high cholesterol, heart disease, and stroke.	1 = at least one of the five conditions present; 0 = none present.	Primary exposure	Included to capture overall burden of cardiometabolic or cardiovascular morbidity in a parsimonious way.

Sex	Self-reported in SHARE.	Categorical variable (specify exact coding used in the analysis, if desired).	Covariate	Included to adjust for demographic differences related to memory and risk-factor distribution.
Age	Self-reported age at interview in SHARE.	Continuous variable in years.	Covariate	Included because memory performance and exposure prevalence vary strongly by age.
Education	Assessed using ISCED-97 in SHARE.	Low (ISCED 0–2), medium (ISCED 3–4), high (ISCED 5–6), and other (unknown or still in education).	Covariate	Included because educational attainment is strongly associated with cognitive performance and may confound exposure–outcome associations.
Activities of daily living (ADL)	Based on reported difficulties in daily self-care activities, including dressing, walking, grooming, eating, transferring in/out of bed, and toileting.	Continuous index ranging from 0 to 6, with higher scores indicating more limitations.	Covariate	Included to adjust for functional status, which may be related to both risk factors and memory.
Enrollment test wave	Obtained from SHARE survey records.	Categorical variable indicating the respondent's enrollment wave.	Covariate	Included to account for study-related differences across waves, such as timing of enrollment and sample composition.

Appendix 5

Supplementary Table S2. Estimated associations and 95% Credible interval (CIs)

Variable	North	West	South	East	Pooled
CMC	-0.19 (-0.27, -0.11)	-0.08 (-0.13, -0.02)	-0.15 (-0.22, -0.09)	-0.08 (-0.18, 0.03)	-0.12 (-0.16, -0.09)
Depression	-1.19 (-1.29, -1.08)	-1.03 (-1.09, -0.97)	-0.39 (-0.46, -0.32)	-0.69 (-0.79, -0.59)	-0.82 (-0.86, -0.77)
Physical inactivity	-0.59 (-0.67, -0.50)	-0.61 (-0.67, -0.55)	-0.44 (-0.51, -0.36)	-0.07 (-0.18, 0.02)	-0.47 (-0.51, -0.43)
Obesity	-0.60 (-0.70, -0.50)	-0.66 (-0.72, -0.59)	0.45 (0.37, 0.53)	0.38 (0.28, 0.48)	-0.16 (-0.21, -0.11)
Living alone	-0.68 (-0.79, -0.58)	-0.54 (-0.60, -0.47)	0.61 (0.53, 0.70)	0.58 (0.47, 0.69)	-0.06 (-0.11, 0.00)

Appendix 6

Supplementary Table S3. Estimated associations and 95% Credible interval (CIs) of education stratification

Variable	Stratum	North	West	South	East	Pooled
CMC	Low	-0.09<br>(-0.24, 0.07)	0.01<br>(-0.09, 0.11)	-0.23<br>(-0.33, -0.13)	-0.01<br>(-0.19, 0.17)	-0.09<br>(-0.15, -0.02)
	Medium/high	-0.29<br>(-0.39, -0.19)	-0.11<br>(-0.19, -0.04)	0.11<br>(-0.01, 0.23)	-0.15<br>(-0.29, -0.01)	-0.09<br>(-0.15, -0.04)
Depression	Low	-1.62<br>(-1.79, -1.44)	-1.23<br>(-1.34, -1.12)	-0.50<br>(-0.60, -0.40)	-0.94<br>(-1.11, -0.78)	-1.04<br>(-1.11, -0.97)
	Medium/high	-1.13<br>(-1.27, -0.99)	-0.89<br>(-0.98, -0.80)	-0.25<br>(-0.39, -0.11)	-0.41<br>(-0.55, -0.27)	-0.68<br>(-0.75, -0.61)
Physical inactivity	Low	-0.83<br>(-0.99, -0.68)	-0.63<br>(-0.73, -0.53)	-0.64<br>(-0.74, -0.53)	-0.26<br>(-0.42, -0.11)	-0.61<br>(-0.68, -0.55)
	Medium/high	-0.51<br>(-0.63, -0.40)	-0.58<br>(-0.66, -0.50)	-0.13<br>(-0.25, -0.01)	0.11<br>(-0.03, 0.25)	-0.33<br>(-0.39, -0.28)
Obesity	Low	-0.72<br>(-0.89, -0.54)	-0.90<br>(-1.01, -0.79)	0.52<br>(0.41, 0.63)	0.38<br>(0.21, 0.55)	-0.24<br>(-0.32, -0.17)
	Medium/high	-0.74<br>(-0.88, -0.60)	-0.59<br>(-0.68, -0.50)	0.30<br>(0.17, 0.44)	0.50<br>(0.36, 0.65)	-0.19<br>(-0.27, -0.12)
Living alone	Low	-1.01<br>(-1.19, -0.83)	-0.64<br>(-0.75, -0.53)	0.66<br>(0.54, 0.78)	0.49<br>(0.30, 0.67)	-0.15<br>(-0.23, -0.07)
	Medium/high	-0.64<br>(-0.79, -0.50)	-0.46<br>(-0.55, -0.37)	0.47<br>(0.32, 0.61)	0.89<br>(0.74, 1.04)	-0.02<br>(-0.10, 0.05)

Appendix 7

Supplementary Table S4. Estimated associations and 95% Credible interval (CIs) of age stratification

Variable	Stratum	North	West	South	East	Pooled
CMC	Younger	-0.34 (-0.46, -0.23)	-0.24 (-0.32, -0.16)	0.06 (-0.04, 0.16)	-0.04 (-0.18, 0.10)	-0.15 (-0.20, -0.09)
	Older	0.02 (-0.10, 0.13)	0.14 (0.06, 0.23)	-0.41 (-0.52, -0.31)	-0.16 (-0.32, 0.00)	-0.10 (-0.16, -0.04)
Depression	Younger	-1.10 (-1.25, -0.96)	-0.88 (-0.96, -0.79)	-0.31 (-0.41, -0.20)	-0.57 (-0.71, -0.42)	-0.71 (-0.77, -0.64)
	Older	-1.27 (-1.42, -1.12)	-1.21 (-1.31, -1.12)	-0.50 (-0.60, -0.39)	-0.91 (-1.06, -0.75)	-0.96 (-1.03, -0.89)
Physical inactivity	Younger	-0.59 (-0.71, -0.47)	-0.64 (-0.72, -0.56)	-0.27 (-0.37, -0.17)	0.09 (-0.05, 0.24)	-0.41 (-0.47, -0.35)
	Older	-0.58 (-0.71, -0.46)	-0.56 (-0.65, -0.48)	-0.65 (-0.75, -0.54)	-0.36 (-0.52, -0.21)	-0.57 (-0.63, -0.51)
Obesity	Younger	-0.65 (-0.80, -0.51)	-0.69 (-0.78, -0.60)	0.36 (0.25, 0.46)	0.33 (0.19, 0.47)	-0.22 (-0.28, -0.15)
	Older	-0.54 (-0.69, -0.38)	-0.61 (-0.71, -0.51)	0.54 (0.43, 0.66)	0.43 (0.26, 0.59)	-0.09 (-0.16, -0.02)
Living alone	Younger	-0.87 (-1.02, -0.72)	-0.62 (-0.72, -0.53)	0.76 (0.63, 0.88)	0.70 (0.54, 0.86)	-0.06 (-0.14, 0.01)
	Older	-0.42 (-0.57, -0.27)	-0.39 (-0.48, -0.30)	0.47 (0.35, 0.58)	0.40 (0.24, 0.56)	-0.02 (-0.09, 0.05)

Reference

1. Tan YV, Roy J. Bayesian additive regression trees and the General BART model. *Stat Med*. 2019;38(25):5048-5069. doi:10.1002/sim.8347

2. Goldstein H. Multilevel mixed linear model analysis using iterative generalized least squares. Biometrika. 1986;73(1):43-56. doi:10.1093/biomet/73.1.43.

3. Chipman HA, George EI, McCulloch RE. BART: Bayesian additive regression trees. Ann Appl Stat. 2010;4(1):266-298.

4. Linero AR, Yang Y. Bayesian regression tree ensembles that adapt to smoothness and sparsity. J R Stat Soc Ser B Stat Methodol. 2018;80(5):1087-1110. doi:10.1111/rssb.12293

5. Linero, A.R.: Softbart: soft bayesian additive regression trees. arXiv preprint arXiv:2210.16375 (2022)

6. Burke DL, Ensor J, Riley RD. Meta-analysis using individual participant data: one-stage and two-stage approaches, and why they may differ. *Stat Med*. 2017;36(5):855-875. doi:10.1002/sim.7141
